# Children’s motivation overcame parental hesitation: active school transportation in Sweden

**DOI:** 10.1093/heapro/day083

**Published:** 2018-10-29

**Authors:** Stina Rutberg, Anna-Karin Lindqvist

**Affiliations:** Department of Health Sciences, Luleå University of Technology, 971 87 Luleå, Sweden

**Keywords:** children, active transport, empowerment, health behavior, parent

## Abstract

To meet the recommendation of 60 min of daily physical activity, children can be encouraged to walk or bike to school, which is known as active school transportation (AST). The aim of this study was to describe parents’ attitudes to AST and to explore their experience when implementing interventions to promote it. To explore parent’s experiences, we collected pre- and post-intervention data via three questionnaires, using both closed and open questioning techniques. The pre-intervention questionnaire informed development of the intervention. Open-ended questions (pre- and post-) were analyzed with qualitative content analysis. In the intervention, there were 42 children, with 63 parents answering pre-intervention questionnaires and 44 answering a post-intervention questionnaire. The analysis resulted in one main theme: children’s motivation and active travel reduces parents’ perception of problems, along with three subthemes: parental concerns and suggestions for solutions, children’s motivation guides parental choice of transport mode, and trying it changes attitudes. In conclusion, it is beneficial to use the enthusiasm and motivation of children to overcome parental hesitation with AST. In addition, it is critical to acknowledge their concerns, as they are the gatekeepers to the children’s use of AST and it is valuable to empower parents when designing relevant interventions. Interventions to increase AST could preferably target changed behavior, and parents’ confidence in their children’s ability to use active transport in a safe and effective way, vs focusing on changing parental attitudes.

## INTRODUCTION

According to the World Health Organization, the recommendation for children’s physical activity is at least 60 min of moderate-to-vigorous intensity daily (WHO, 2010). However, many children around the world do not reach the recommended levels (Hallal *et al.*, 2014). To increase daily physical activity, children could be encouraged to walk or bike to school, which is known as active school transportation (AST). AST is an important focus of research and policy attention, as it incorporates health, environment and transport safety: a shift to a more active mode of transport would yield several benefits in all three areas (Badland and Schofield, 2005). 

Unfortunately, there has been a decline in AST in many countries (Rothman *et al.*, 2017b). The only country to achieve the highest grade of A (more than 80% of the children) for AST, with 38 countries participating in the Global Physical Activity Matrix, was the Netherlands (Burghard *et al.*, 2016), while Sweden achieved a grade of C+ (between 41 and 60% of the children) (Nyström *et al.*, 2016). The decline is problematic, because when an increasing number of parents drive their children to school, the surrounding environment becomes a dangerous place for bicyclists and pedestrians (Rothman *et al.*, 2017a), that is, children become less physically active (Faulkner *et al.*, 2009) and air pollution also increases (Gauderman *et al.*, 2015). Moreover, the Organization for Economic Cooperation and Development (OECD) suggests that an increase in active transport could be an effective way of promoting health and preventing obesity (Sassi, 2010). Nevertheless, children’s AST is likely to further decline in the absence of interventions to increase it (Pabayo *et al.*, 2011). The number of children using AST is influenced by attitudes, as well as social and personal factors (Panter *et al.*, 2010), whereas parents’ attitudes and behavior are especially crucial (Mah *et al.*, 2017). Previous studies report that parents of young children refer to traffic and personal safety as strong barriers to using AST (Brunton *et al.*, 2006). Furthermore, both the children’s and parent’s self-efficacy (i.e. parents’ belief in the child’s competence to safely use AST) affects the mode of transport, even though parental self-efficacy has the most impact (Lu *et al.*, 2015). Panter *et al.*(Panter *et al.*, 2010) found that positive attitudes, social support and encouragement, and a sense of traffic and safety were correlated with greater use of AST. 

Since parents are the main gatekeepers of AST, exploring their experience and attitudes about AST is invaluable, such that they should be involved in designing AST interventions. The European Commission’s (EU) Health Strategy ‘Together for Health’ recommends an investment in people’s health, particularly through empowerment (Young *et al.*, 2013); the involvement of end-users has been suggested to improve the efficiency of AST intervention (Mah *et al.*, 2017). Panter *et al.* (Panter *et al.*, 2010) stated that changing parental perceptions is an important strategy for promoting AST; however, it could be worthwhile to try to change behavior to increase AST, instead of putting effort into changing parental attitudes (Kroesen *et al.*, 2017). Further research is needed to develop effective interventions to promote AST, given the complexity of the individual, social and environmental factors influencing choice of travel to school. The aim of this study was to describe parents’ attitudes to AST and explore their experiences when implementing an intervention to promote it.

## METHODS

### Design

This is one of the three studies (Lindqvist and Rutberg, 2018; Rutberg and Lindqvist, 2018) exploring an empowered- and gamification-inspired program for children’s AST. The first study presents the intervention and theory underpinning it. The second study presents teacher’s and students’ experiences while participating in the intervention. This pilot study used an emergent design, and research procedures evolved simultaneously in response to earlier results of the research (Morgan *et al.*, 2008). To explore parent’s attitudes and experiences, we collected data pre- and post-intervention, with two questionnaires including both open and closed questions. The pre-intervention data were used to design the intervention that was later evaluated in the post-intervention data collection (Rauscher and Greenfield, 2009).

### Procedure and participants

Information about the project was shared with the principals of 29 elementary schools, grades 1–3, in a municipality of northern Sweden. Five schools were interested in participating and expressed their motivation to be included in the pilot study. One school was chosen based on having had extensive problems with traffic, caused by parents driving their children to and from school. The principal and two teachers gave their informed consent to participate in the study. The characteristics of the municipality and participants are shown in Table 1. The study was approved by the Regional Ethical Board.

**Table 1:** Characteristics of the municipality, children, and parents participating in the study

The municipality	Approximately 80 000 inhabitants situated in the northern part of Sweden.
The primary school	270 children; the school is situated in a neighborhood with apartment buildings and detached houses.
	The distance to school varied between 0.2 and 6.0 km with an average of 1.3 km.
The children	42 children (23 boys and 19 girls), aged 7–8 years participated in the program.
The parents	63 parents (35 women and 26 men) answered the pre-intervention questionnaire.
	44 parents (26 women and 18 men) participated in a qualitative second data collection after the intervention.
	73% of the participants had a college/university education, while 27% had an upper secondary education.
	97% were Swedish citizens.
	44% were parents of girls and 56% were parents of boys.
	52% answered that their children sometimes used AST and 21% answered that their children used AST every day.

### The intervention and data collection

Building on the constructs of Social Cognitive Theory (Bandura, 2004), the program included activities that increased knowledge, enhanced perceived self-efficacy among parents and children, and used an array of facilitators and impediments for change (Lindqvist and Rutberg, 2018).

#### Pre-intervention

The program was initiated with a parental meeting, with discussions about the advantages and problems of AST; this was geared to increase parents’ knowledge and motivation, 3 weeks before the AST period. The parents completed a questionnaire concerning intrapersonal psychological factors, known as the Modified Integrated Model for Children’s Active Commuting to School, which was shown to have a good model fit and could thereby enable health behavior researchers to design effective interventions to promote AST (Lu *et al.*, 2014). The questionnaire was translated into Swedish, according to principles of good practice for cultural adaptation (Wild *et al.*, 2005). In this process, two items about perceived issues (crossing guards) and use of self-efficacy (despite it being hot outside) were eliminated. In addition, the parents answered an open letter, which was introduced with the text: ‘You have answered a questionnaire concerning AST, including obstacles your child might experience while using AST. Please describe how these obstacles can be overcome’. The results were used to further develop the intervention; for example, parents were concerned with letting their children go to school alone, so the program included dividing the children into small groups to make both walking and biking safer.

#### In-class intervention

The in-class intervention started with workshops for the children 1 week prior to the start of AST. The workshops aimed at increasing their knowledge and motivation with three themes: (i) health, (ii) traffic safety and (iii) environment. During 4 weeks of AST, the children were given weekly assignments and they measured their use of AST. The assignments were designed by the teachers and were connected to the curriculum: for example, counting the people they met on their way to school or looking for traffic signs. The measurements of AST became a joint progress bar of class effort, and a motivator for success, which were also used in math and geography lessons. These gamification elements were used to inspire internal motivation for engaging in healthy behavior (Deterding *et al.*, 2011). The recurrent assignments earned the children badges, and moving to the next challenge.

#### Post-intervention

Two weeks after ending the intervention, a second open letter was introduced with four questions; ‘How have you as a parent experienced your child’s participation in the AST project’; ‘If this project was used in a different class, is there something we should do again, and are there things that should be changed’; ‘What was your attitude towards AST before the intervention, and have your attitudes changed after participating’; and ‘Has your own choice of travel modes changed during the project?’

### Data analysis

The collected data on the closed questions in the pre-intervention questionnaire were analyzed and presented with descriptive statistics. The qualitative data from the two open letters were analyzed as a whole with qualitative content analysis, inspired by Graneheim and Lundman (Graneheim and Lundman, 2004), with both authors actively participating in the following procedure: (i) written material was first read several times to obtain a sense of the overall data; (ii) the text was divided into meaning-units; (iii) during the abstraction process, the meaning-units were coded, and the codes were then compared, contrasted, and sorted into preliminary categories, with the authors striving to stay close to the text throughout the steps; (iv) the preliminary categories were sorted; and (v) the categories were sorted into one main theme and three subthemes. Quotations were used to strengthen the credibility of the study.

## RESULTS

The pre-intervention questionnaire showed that almost half of the parents had concerns about traffic-related issues and more than half had concerns about violence or crime in letting their children use AST. Moreover, lack of company was perceived as a problem for almost half of the parents; parents’ self-efficacy in their children showed that 62% of them were very sure their child could use AST with friends or classmates, although 13% were not sure it would be possible. About two-thirds of parents believed their child would be healthier and ready to learn in school with involvement in AST. The questionnaire results are shown in Table 2.

**Table 2:** Parents’ perceived concerns about AST (10 items), parents’ self-efficacy in regards to their children (13 items), and parents’ beliefs and outcome evaluations (7 items)

Perceived concerns	Not a problem	Sometimes	Always
Which problems have affected your decision to allow your child to walk or bike to and from school:			
Distance	68%	21%	11%
Time	70%	20%	10%
Speed of traffic	51%	28%	21%
Amount of traffic	53%	26%	21%
Lack of company (adults and children)	52%	30%	18%
Lack of sidewalks	70%	22%	8%
Safety at crossings	39%	25%	36%
Violence or crime	44%	36%	20%
Weather or climate	73%	24%	3%
Stray or dangerous animals	88%	9%	3%
Self-efficacy	Not sure	A little sure	Very sure
I am sure that I can allow my child to walk to and from school:			
Even if we live far from school	45%	34%	21%
Even if there is a lot of traffic	53%	34%	13%
Even if it is cold outside	21%	17%	62%
Even if it is raining outside	10%	22%	68%
Even if the other children don’t walk	25%	38%	37%
Even if I cannot walk with my child	38%	26%	36%
Even if I have worries or problems	18%	26%	56%
Even if I can drive my child	18%	22%	60%
At least once every week	12%	26%	62%
Every day of the week	28%	26%	46%
With me	9%	12%	79%
With my child’s friends or classmates	13%	25%	62%
Alone, without other children or adults	43%	28%	29%
Beliefs and outcome evaluations			
If my child uses active school transportation:			
My child will be healthier	10%	20%	70%
My child will get more physical activity	6%	8%	86%
My child will not become overweight	21%	41%	38%
My child will cross streets safely	33%	42%	25%
My child will be ready to learn in school	5%	28%	67%
My child will be on-time for school	8%	40%	52%
I will have more time for other things	39%	41%	20%

### Children’s motivation and active travel reduces parents’ perception of problems

The qualitative analysis revealed a complex picture of parents’ attitudes to AST when using an intervention to promote it. The first subtheme ‘Parental concerns and suggestions for solutions’ shows their concerns and fears, as well as solutions to it. When the parents contributed to the design of the intervention, their sense of security increased. Moreover, the second subtheme ‘Children’s motivation guides parental choice of transport mode’ shows that children’s enthusiasm to use AST, including gamification and increased knowledge, helped parents agree on the mode of active travel. The third subtheme ‘Trying it changes attitudes’ shows that when the parents found the use of AST to be safe, it positively affected their attitudes.

#### Parental concerns and suggestions for solutions

The parents described pre-intervention worries of letting their child walk or bike to school, mainly due to being too young or not ready to manage the responsibility. Parents’ concerns predominately surrounded traffic safety and ‘stranger danger’. Infrastructure, such as lack of pedestrian crossings and alternative ways to get across the road were described as contributing factors to their concern with AST. One parent commented that even if the entire way to school was safe, one risky crossing could be enough to not let the child use AST. The parents offered improvement suggestions in the form of speed barriers, more crossing points in the area, and better walking and cycling lanes. In addition, enhanced traffic understanding among children was discussed as a way of increasing safety, and reassurance that the school would inform the parents if the child did not arrive at the school or get there on time; doubts about this made them hesitate to let the children go by themselves without an adult.

Parents felt that if their child had company on the way, it would be easier to relax about letting them use AST. Post-intervention, parents commented that the children appreciated going with friends, and that as parents that created more security. Some communication problems created issues about arrangements (e.g. how a small group should accompany each other), but the parents solved that. One parent acknowledged that two children in the pair used different ways of getting to school: his son was told to use a route that was slightly longer, but avoided dangerous traffic, yet his friend took the shortest way.

*It's been a good thing to get the kids to go together to school. The only issue is the uncertainty if you send children without a parent is to know if they have arrived or not. Still, it has been very positive and I feel safe to let my daughter use AST.*

Parents commented on reasons for pre-intervention, beyond safety, for not using AST. Using it required more planning: for example, clothing in terms of the weather. Moreover, they thought it was difficult to manage AST when younger siblings were at daycare at the same time. In addition, using AST meant negotiating advantages vs disadvantages, such as taking the time to follow the child when there were many things to carry to school and thus take in the car. One parent said that using AST would probably mean less time at work, but it would be worth it. Parents also said that it was more comfortable to take the car in the winter, when it was cold. In addition, some parents had concerns of losing daily contact with the children’s teachers if they did not accompany them to school.

*I want to follow my child partly for the traffic in the area, partly because it is too early to let go of the dialogue with teachers. It is often the only chance to get feedback or to get answers to questions. This means that you sometimes have to take the car.*

#### Children’s motivation guides parental choice of transport mode

Post-intervention, it was evident that children’s motivation to use AST affected their parents’ choice of allowing it. The parents stated that both elements, gamification as well as increased knowledge, made their children enthusiastic to use AST. One parent commented that before attending the meeting, she had made up her mind about her child participating in the project.

*My girl was so persistent about me agreeing to her using AST during the project, that I told her I would agree to it without even knowing what it was about.*

Parents reported how their children thought it was fun to put stickers on the board for every kilometer they walked or biked to and from school; they were trying to meet the challenge and were motivated to use AST. The children also inspired the parents to let them use AST more often; many parents expressed that not even bad weather created an issue about using active transport. They expressed that their children were proud of being part of this project; for example, they were proud of how many kilometers they had accrued. Halfway into the intervention, they received a cap with the text ‘I walk and bike to school’ (in Swedish), which served as an acknowledgement of being part of the project. This made them engage in more walking and biking, and also made the parents feel more positive.

*Very positive! Previously, it could be an argument about going by car to school when there was bad weather, but during the project, it has been a matter of course to ride a bike even though the weather was bad.*

According to the parents, the project also educated the children; furthermore, AST had become a matter of conversation at home. They learned about the body and why physical activity is healthy, and about traffic safety and its effect on the environment. Learning was highlighted as an important aspect, since it was expected to facilitate change for the future.

*My child has reflected over transport in general, which never otherwise had happened. It has given my daughter a will and an understanding of why it is good to be physically active.*

Moreover, parents themselves used more active transport during the project. Some of them already used active transport or had started to use it more frequently. Some parents stated that they developed zero tolerance to using a car, while some commented on the necessity to be good role-models and that this project increased the degree of active transport; thus, encouragement was important from the school as well as from the parents.

*Positive for the whole family that he cycled every day, which also made me go by bike more often. If you hesitate between the bike and the car, it is now more likely that I will choose the bike.*

#### Trying it changes attitudes

Almost all parents were more positive to AST after the project than before; those who were already positive and used AST regularly expressed a more positive attitude to maintain it. Some expressed doubts about letting their child walk or bike to school, but when they saw that everything went well, this concern changed somewhat. Parents described positive experiences with AST, as it was a good starting point to being outside and active, along with saving time and how it was easier to be on time. All children using AST continued with it.

*I was skeptical before, and felt like the study would force us to change our way of managing transport to school, but afterwards, when I realized that she managed to go to school by herself with a friend, then it was only positive, in addition to having saved time in the mornings, and I am very glad that you did this.*

However, not all parents felt confidence in letting their child go to school without adults. One parent stated that he thought children were not mature enough to go to school without an adult, while some thought it would have been more appropriate to start this project at age 8 or older. In addition, one parent wrote that not all children at age 7 have a key to the home or should leave school to return home alone.

By letting their children use AST, most parents acknowledged that they had the competence to get safely to school, which reflected their children’s development. They had seen that their child had grown with the task, as one parent talked about how this experience made her child take more responsibility and become more mature. Another parent said:

*My kid can now go to school without us accompanying. She knows where she should meet with her classmate, how to contact each other if there is a change of plans. The best thing I think is that she gained confidence and now likes to ride her bike very much. Sometimes she even goes back and forth between city central and the school.*

## DISCUSSION

The focus of this study was to describe parents’ attitudes to AST and explore their experience when promoting it. The results indicate that children’s motivation, inspired by gamification elements and active travel, reduces parents’ perception of AST barriers. Empowerment was a major component in this intervention, with previous research showing how it enhances implementation and contributes to greater compatibility with user needs (Flay *et al.*, 2005); this in turn increases the likelihood that programs will be sustained (Durlak and DuPre, 2008).

The questionnaire on parental psychological factors was the starting point for the project, as this input was used to design interventions. The questionnaire demonstrated that the parents’ perceived concerns about AST included speed and degree of traffic, as well as a lack of company, a result which was consistent with previous research (Shaw *et al.*, 2015; Ahern *et al.*, 2017). By accommodating the parents’ wishes for their children to have company on their way to school, we saw that it was possible to start using AST without solving all of the problems parents perceived. We considered the use of walking and school buses (with parents accompanying the children); however, previous research showed that this approach had low sustainability (Ahern *et al.*, 2017) and therefore this intervention was built on children accompanying children. As the results show, two-thirds of parents in the pre-intervention questionnaire were confident their children could get to school with a friend, with only a few parents in the post-intervention questionnaire mentioning that walking alone could be a problem. This would indicate that this choice might be feasible for many children.

Our findings show that parents’ belief in children’s competence to manage AST increased. This is relevant, as there is evidence that children’s independent mobility is declining with significant consequences for general health, and physical, social, and mental development (Shaw *et al.*, 2015). It has been suggested that allowing children increased levels of independence in their travel becomes an important opportunity to develop and consolidate skills on how to strengthen their independent mobility (Shaw *et al.*, 2015). Furthermore, when children’s mobility is limited, this can affect many of the opportunities to demonstrate their capabilities to their parents (Ahern *et al.*, 2017).

This study also reveals that when parents experienced how their children could use AST safely, they altered their attitudes and became more positive about it. This finding is consistent with Bandura’s theory, which includes a belief in an individual’s ability to achieve a goal, altered by direct mastery experiences (Bandura, 2004). Our result could also be interpreted as how active travel behavior affects attitudes, and research shows that the effects of active travel on these attitudes are as efficient as the opposite (Zuniga, 2012; Kroesen *et al.*, 2017). Nevertheless, as reported in our previous study (Rutberg and Lindqvist, 2018), the children who participated in the intervention were self-efficacious from the beginning, and most of them expressed no fear about walking or bicycling to school.

Our findings indicate that regular use of AST can diminish parents’ perception of barriers and concerns, which were raised pre-intervention; this can be interpreted that it is more important to focus on increasing the use of AST instead of targeting altered attitudes. This is also consistent with Festinger’s theory of cognitive dissonance (Festinger, 1962). Cognitive dissonance refers to a situation involving conflicting attitudes, beliefs or behaviors, which produce a feeling of discomfort; this leads to an alteration of these attitudes and beliefs to reduce discomfort and restore balance (Festinger, 1962). Our findings can be compared with those of Seraj *et al.* (Seraj *et al.*, 2012), who showed that parents using active transport or whose children used AST had less concerns about crime, weather, volume of traffic and distance to school—compared with parents using motorized transport.

One limitation of this study is that it was performed in a small-town school, which may have influenced parents’ beliefs and opinions. Therefore, the findings might not be transferable to schools in areas where the road environment is less supportive to safe AST. Due to the complex interactions between socioeconomic, environmental and cultural factors, further research is needed, as it is important to consider these factors when designing effective programs to promote children’s use of AST. Future research should include a wider target group that will assist in accounting for these factors.

## CONCLUSION

This study shows how beneficial it is to seize children’s enthusiasm and motivation to overcome parental hesitation with AST. However, one must acknowledge parental concerns, as they are the gatekeepers of the children’s use of AST, and it is valuable to empower parents when designing pertinent interventions. The idea of children being accompanied by other children prompted a shift in behavior. Moreover, our findings suggest that interventions to increase AST should target changing behavior and parental self-efficacy in their children’s ability to safely use active transport, instead of focusing on changing parental attitudes.
